# Platelet-Rich Plasma (PRP) and Recombinant Growth Factor Therapies in Cutaneous Wound Healing: Mechanisms, Clinical Applications, and Future Directions

**DOI:** 10.3390/jcm14238583

**Published:** 2025-12-03

**Authors:** Abu-Bakr Ahmed, Spencer Thatcher, Joshua Khorsandi, Zahra Ahmed, Michael Lee, Adam Jaouhari, Braydon Bond, Aftab Merchant

**Affiliations:** 1 Kirk Kerkorian School of Medicine, University of Nevada, Las Vegas, Las Vegas, NV 89106, USA; ahmeda15@unlv.nevada.edu (A.-B.A.); thatcs1@unlv.nevada.edu (S.T.); khorsand@unlv.nevada.edu (J.K.); leem72@unlv.nevada.edu (M.L.); 2 Lake Erie College of Osteopathic Medicine, Elmira, NY 14901, USA; zahmed75797@med.lecom.edu; 3 School of Integrated Health Sciences, University of Nevada, Las Vegas, Las Vegas, NV 89106, USA; jaouhari@unlv.nevada.edu (A.J.); bondb1@unlv.nevada.edu (B.B.); 4 Department of Medical Education, Kirk Kerkorian School of Medicine, University of Nevada, Las Vegas, Las Vegas, NV 89106, USA

**Keywords:** platelet-rich plasma, recombinant growth factors, platelet-derived growth factor (PDGF), epidermal growth factor (EGF), basic fibroblast growth factor (bFGF), vascular endothelial growth factor (VEGF), wound healing, chronic ulcers, diabetic foot ulcers, regenerative medicine

## Abstract

**Background**: Chronic cutaneous wounds such as diabetic foot ulcers, venous leg ulcers, pressure injuries, and burns remain a global clinical burden. These wounds are often arrested in inflammatory or ischemic stages due to impaired angiogenesis and growth factor deficiencies. Biologic therapies, such as platelet-rich plasma (PRP) and recombinant growth factors, aim to restore these deficits and accelerate repair. **Methods**: A narrative review of PubMed and Google Scholar (2015–2025) identified 64 English-language studies, including randomized controlled trials, meta-analyses, and translational investigations evaluating PRP and recombinant growth factors in wound healing. **Results**: Randomized trials and meta-analyses show that adjunctive autologous PRP increases complete wound closure versus standard care in chronic ulcers, including diabetic foot and venous leg ulcers (odds ratios ≈ 2–8), and improves healing rates in pressure injuries (odds ratio ≈ 3.4), without increasing adverse events. In diabetic foot ulcers, PDGF-BB and EGF, together with PRP, consistently improve complete healing and reduce ulcer area. In burns, topical EGF and bFGF shorten healing time by ~3 days in superficial partial-thickness wounds and by >5 days in deeper burns, with generally improved scar outcomes. **Conclusions**: PRP offers broad, autologous biologic activation, while recombinant growth factors deliver high-potency, targeted precision. Together, they represent complementary regenerative strategies that can shorten healing times and improve outcomes in chronic wounds. Standardized multicenter trials quantifying cytokine composition, cost-effectiveness, and long-term limb-salvage benefit are warranted to guide their integration into routine clinical practice.

## 1. Introduction

Chronic cutaneous wounds affect millions of patients worldwide and represent a “silent epidemic” in healthcare [1,2]. Diabetic foot ulcers (DFUs), pressure injuries, venous leg ulcers, and other hard-to-heal wounds are characterized by prolonged inflammation, impaired angiogenesis, and deficits in growth factors at the wound site [3,4]. These pathological wounds often fail to progress through normal healing stages, resulting in persistent open ulcers that diminish quality of life and increase risks of infection and amputation [2,4]. Standard wound care, such as offloading, debridement, infection control, and advanced dressings, is essential but sometimes insufficient to achieve closure in chronic wounds. There is thus great interest in adjunctive biologic therapies that actively stimulate the healing process.

Platelet-rich plasma (PRP) and recombinant growth factor products have emerged over the past decade as two major classes of regenerative therapies for wound healing. PRP is an autologous blood-derived preparation enriched with platelets and their associated growth factors and cytokines [5]. When applied to wounds (typically as a topical gel or injected preparation), activated platelets release a concentrated milieu of signaling molecules, such as platelet-derived growth factor (PDGF), transforming growth factor-β (TGF-β), vascular endothelial growth factor (VEGF), epidermal growth factor (EGF), and others, which can promote cell migration, angiogenesis, and extracellular matrix deposition to accelerate repair [6]. Recombinant growth factor therapy, on the other hand, involves delivering a specific bioengineered protein important to wound healing at pharmacologic doses directly to the wound. The rationale is to overcome local growth factor deficiencies by supplementing key signaling molecules that drive granulation tissue formation and re-epithelialization [4].

Early clinical studies of topical growth factor proteins in the 1990s showed promise, leading to the first (and to date only) FDA-approved drug for wound healing: becaplermin (recombinant human PDGF-BB) gel for diabetic neuropathic foot ulcers [7]. In the years since, dozens of clinical trials have explored PRP and various growth factor treatments in chronic wounds, with some demonstrating improved healing outcomes [2,5]. Results, however, have been mixed. Systematic reviews show that many studies are small or carry some form of bias, making it difficult to draw accurate conclusions from them [2,8]. Reflecting the current state of research, a 2023 network meta-analysis concluded that the evidence for EGF, PRP, and PDGF in DFU healing is still considered low-quality. Consequently, international guidelines advise caution against their routine use, pending stronger data [2].

This comprehensive review consolidates recent findings on biologic therapies for wound healing, focusing on platelet-rich plasma and recombinant growth factors. We examine the molecular mechanisms of action, assess comparative therapeutic efficacy, and describe applications for diverse wound etiologies. The analysis extends to safety, cost-effectiveness, and the challenges of clinical translation. The review concludes by highlighting current evidence gaps and proposing key directions for future investigation. This synthesis is pertinent given the recent proliferation of high-level evidence from meta-analyses and randomized trials, which has renewed focus on biologic solutions for chronic wound care.

## 2. Materials and Methods

This work was designed as a narrative review with the objective of synthesizing mechanistic and clinical evidence on platelet-rich plasma (PRP) and recombinant growth factor therapies for cutaneous wound healing. The scope was restricted to interventions applied to cutaneous wounds (e.g., diabetic foot ulcers, venous leg ulcers, pressure injuries, burns, and acute surgical or traumatic wounds) and to preclinical models with clear translational relevance to skin repair. Evidence was organized thematically into (i) molecular and cellular mechanisms; (ii) clinical effectiveness across wound types; and (iii) safety, cost, and implementation considerations, and was synthesized narratively rather than through formal meta-analysis or comparative-effectiveness ranking.

We searched PubMed (https://pubmed.ncbi.nlm.nih.gov) and Google Scholar (https://scholar.google.com) for English-language articles published from 1 January 2015 through 25 July 2025. Seminal pre-2015 mechanistic and clinical studies identified through citation tracking were also included when directly relevant to cutaneous wound healing. In PubMed, we used two broad strategies: one targeting PRP and one targeting recombinant growth factors, both in the context of wound healing. Search strings included (“platelet-rich plasma” OR PRP OR “platelet concentrate”) AND (“wound healing” OR “chronic wound*” OR ulcer* OR “diabetic foot” OR “venous leg ulcer*” OR “pressure injur*” OR burn*), and (becaplermin OR “recombinant PDGF” OR “platelet-derived growth factor” OR “epidermal growth factor” OR “basic fibroblast growth factor” OR “vascular endothelial growth factor”) AND (wound* OR ulcer* OR burn*). Searches were last updated on 25 July 2025. Google Scholar was used as a supplementary source, primarily for forward and backward citation tracking and to identify additional reviews. Due to its ranking algorithm being proprietary and less reproducible than PubMed, Google Scholar results were not treated as a stand-alone systematic search.

Two authors independently screened titles and abstracts of all retrieved records, followed by a full-text review of potentially eligible articles. We included human clinical studies (randomized controlled trials, non-randomized trials, cohort and case–control studies, and case series) evaluating PRP or recombinant growth factor therapy as part of wound management; systematic reviews, meta-analyses, and network meta-analyses focused on these interventions; and in vivo preclinical studies with mechanistic or translational relevance to cutaneous wound healing. Exclusion criteria were: non-cutaneous wound models, in vitro-only studies without in vivo or clinical components, studies focused solely on surgical or device technique without PRP or growth factor therapy, narrative commentaries without original data, conference abstracts lacking full-text articles, and non-English publications. Disagreements were resolved by discussion and, when needed, consultation with a senior author. Across databases, we screened 877 records in total (PubMed: 453 articles (182 for PRP and 271 for recombinant growth factors); Google Scholar: 424 results (221 for PRP and 203 for recombinant growth factors)). After title/abstract screening and full-text assessment, 64 publications met the inclusion criteria and were retained for detailed synthesis.

Given the narrative design of this review, we did not perform formal risk-of-bias scoring using standardized tools. Instead, when summarizing clinical outcomes, we qualitatively appraised each study’s robustness, including sample size, randomization and blinding procedures, completeness of follow-up, and clarity of outcome definitions, and we highlight these factors in the Discussion and Limitations. For preclinical and mechanistic studies, we considered the biological model, dosing and delivery of PRP or growth factor, and relevance of outcome measures to human cutaneous wound repair. Because the evidence base spans heterogeneous study designs (RCTs, observational cohorts, case series, animal models, and network meta-analyses), quantitative estimates such as odds ratios and rank probabilities are reported descriptively from the original studies and should be interpreted cautiously in light of this heterogeneity and potential risk of bias, rather than as definitive comparative-effectiveness rankings.

## 3. Discussion

**Figure 1:**
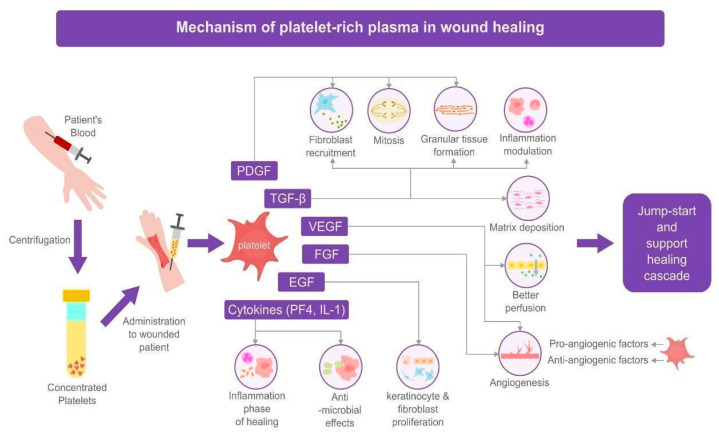
Mechanism of action of platelet-rich plasma in wound healing.

**Figure 2:**
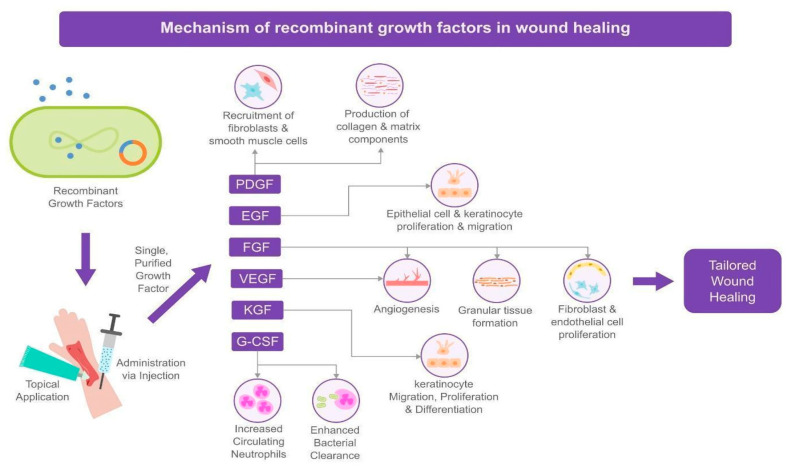
Summary of the mechanism of recombinant growth factors in wound healing.

Wound healing proceeds through four overlapping biological phases: hemostasis, inflammation, proliferation, and remodeling. During the hemostatic phase, platelets aggregate to form a fibrin clot that acts as a temporary matrix. The inflammatory phase recruits leukocytes and cytokines to clear debris and pathogens, followed by the proliferative phase, in which fibroblasts, endothelial cells, and keratinocytes drive granulation and re-epithelialization. Finally, remodeling organizes collagen and restores tensile strength. Chronic wounds often stall in the inflammatory stage due to growth factor deficits and impaired angiogenesis. Therapies such as PRP and recombinant growth factors aim to reactivate or accelerate these physiologic phases. This framework underpins the mechanistic rationale for the biologic interventions described below. Figure 1 and Figure 2 schematically map how PRP and key recombinant growth factors interface with these phases of repair, providing visual anchors for the mechanistic discussion that follows.

### 3.1. Mechanisms of Action of PRP and Growth Factors

#### 3.1.1. Platelet-Rich Plasma (PRP)

PRP is typically prepared by centrifuging a patient’s blood to concentrate platelets in a small volume of plasma. By definition, PRP contains a higher platelet count than baseline whole blood (often 2–9× depending on preparation) [9]. Platelets are reservoirs of growth factors stored in alpha granules, and upon activation (e.g., by thrombin, calcium, or collagen exposure), they release a burst of signaling proteins and cytokines (Figure 1). Key growth factors delivered by PRP include PDGF (a potent mitogen and chemoattractant for fibroblasts and smooth muscle cells), TGF-β (which modulates inflammation and stimulates matrix deposition), VEGF and basic FGF (which promote angiogenesis), and EGF (which stimulates keratinocyte and fibroblast proliferation) [6]. Through these factors, PRP can theoretically recapitulate several aspects of the body’s natural wound healing signals in a concentrated form.

Mechanistically, PRP’s multifactorial cocktail acts on multiple cell types involved in repair. PDGF and TGF-β from platelets recruit fibroblasts and support granulation tissue formation, while VEGF and FGF enhance the sprouting of new blood vessels into the wound bed (angiogenesis) [10,11]. EGF and VEGF contribute to re-epithelialization by encouraging keratinocyte migration and by improving perfusion, respectively [4,12]. PRP also contains platelet-derived cytokines like platelet factor 4 and interleukin-1, which can augment the inflammatory phase of healing in a beneficial way and potentially have antimicrobial effects. Notably, platelets carry both pro-angiogenic and anti-angiogenic factors in separate granules, and the net effect may depend on activation context [11]. Overall, the application of PRP to a wound is thought to jump-start the healing cascade by providing a provisional fibrin matrix (if used as a gel or clot) and a rich supply of growth factors that accelerate cell proliferation and tissue regeneration [6]. Preclinical studies show PRP can stimulate stem cell activity and collagen deposition in wounds [5].

It should be noted that PRP preparations are not uniform, and their mechanism can vary with composition [13]. Some formulations include leukocytes (leukocyte-rich PRP), which add white-blood-cell-derived cytokines and potential antimicrobial activity but may also accentuate local inflammation [14,15]. Other preparations are leukocyte-poor. PRP can be delivered either as a liquid for injection or as an activated clot/gel for topical use. Upon activation, it polymerizes into a fibrin matrix that functions as a three-dimensional scaffold for cellular infiltration [15,16]. Despite these variables, the unifying mechanism is the provision of a supraphysiologic local concentration of the patient’s own regenerative signals effectively “turbo-charging” physiologic healing pathways that are often inadequate in chronic wounds [13]. Taken together, these actions show how PRP delivers, in a single autologous preparation, many of the same pro-angiogenic, pro-granulation, and pro-epithelial signals that are later targeted individually by recombinant growth factor therapies (Figure 1).

#### 3.1.2. Recombinant Growth Factors

Recombinant growth factor therapy involves the exogenous application of a specific human growth factor produced via recombinant DNA technology, usually in a topical gel/cream or injectable form. By delivering a single, purified signaling protein at a high concentration, this approach targets distinct receptor-mediated pathways known to be critical for wound healing. Several growth factors have been studied in this context.

Among these, platelet-derived growth factor (PDGF) is the most extensively studied and clinically validated recombinant growth factor in wound healing. PDGF is a family of dimeric proteins (AA, BB, CC, DD isoforms) that bind PDGF receptors on cells. The PDGF-BB homodimer (becaplermin) is the form used clinically. PDGF is released early after injury (by platelets, macrophages, etc.) and has broad mitogenic and chemotactic effects [17]. It recruits fibroblasts and smooth muscle cells into the wound, and stimulates them to synthesize collagen and other matrix components. PDGF also promotes endothelial cell proliferation, contributing to angiogenesis, and it secondarily triggers production of TGF-β and IGF-1, enhancing matrix deposition and epithelialization [17]. Clinically, recombinant PDGF has been shown to “jump start” chronic ulcers: it significantly increases the rate of complete healing in diabetic foot ulcers, as evidenced by multiple RCTs and meta-analyses [2]. It is effective, particularly in neuropathic ulcers, when combined with good wound care [2]. PDGF is less effective in ischemic wounds (unless blood supply is restored) since cells cannot respond if perfusion is poor. Becaplermin requires once-daily topical application and a moist wound environment; it has demonstrated an ~1.5–2.0 fold increase in healing rate of DFUs in controlled trials [2,18]. Notably, PDGF is the only growth factor with regulatory approval for wound healing indications [18].

In addition to PDGF, another well-studied recombinant growth factor in wound healing is epidermal growth factor (EGF), which plays a particularly important role in epithelial regeneration and surface repair. EGF is primarily a driver of epithelial cell and keratinocyte proliferation and migration. It binds the EGF receptor (EGFR/HER1) on keratinocytes, fibroblasts, and endothelial cells, activating pathways that lead to cell division and migration. EGF’s role in wound healing includes promoting re-epithelialization (closure of the wound surface) and contributing to angiogenesis and granulation tissue formation [17]. Recombinant human EGF has been developed as a therapy (for example, as intralesional injections for DFUs). Clinical studies, including large trials in diabetic foot ulcers, have shown that EGF can dramatically improve healing rates in recalcitrant ulcers, even achieving full granulation in wounds otherwise deemed unlikely to heal [2,19,20]. A network meta-analysis found that EGF therapy was associated with the highest likelihood of complete healing among various growth factors tested in DFUs [19]. It has been effectively used in some countries as an injectable around wound edges to stimulate tissue growth. Partial-thickness burns and donor site wounds have also responded to topical EGF with faster epithelial coverage [20]. EGF is generally well tolerated. Because it is a naturally occurring human protein, systemic effects are minimal when used locally.

While EGF primarily facilitates epithelial resurfacing, fibroblast growth factors (FGFs) play a broader role in both stromal and vascular repair processes during wound healing. FGFs are a family of growth factors; basic FGF (bFGF or FGF-2) and acidic FGF (FGF-1) are most relevant in wounds. FGFs promote the proliferation of fibroblasts and endothelial cells, thus playing a significant role in granulation tissue formation and angiogenesis. FGF also indirectly supports epithelialization by inducing keratinocyte growth factors like TGF-α [17]. In Japan, a topical recombinant bFGF spray (trafermin) has been used clinically for chronic ulcers, and studies report enhanced healing with FGF treatment, especially in pressure ulcers and burns (where it can speed up granulation over exposed bone/tendon) [21]. Preclinical models in diabetic mice have shown that bFGF application accelerates wound closure [21]. One interesting aspect is FGFs’ effect on scarring: there is some evidence that FGF therapy may reduce hypertrophic scarring by modulating fibroblast activity [21].

In parallel with cellular proliferation and matrix formation, successful wound healing also depends on the restoration of vascular supply, a process predominantly orchestrated by vascular endothelial growth factor (VEGF). VEGF is the master regulator of angiogenesis. It is normally upregulated in wounds to stimulate new blood vessel sprouting from existing vessels, ensuring that oxygen and nutrients can reach the healing tissue [17]. Delivering VEGF to wounds aims to overcome ischemia/hypoxia in poorly perfused tissue. Experimental delivery of VEGF (protein or gene therapy) has shown improved angiogenesis and wound closure in models of diabetic ulcers and in ischemic wounds [22]. However, the clinical use of VEGF protein is limited by its short half-life and potential for promoting unstable or leaky vessels; gene therapy approaches (plasmid or viral delivery of VEGF gene) have been tried in clinical trials with mixed results [22]. No recombinant VEGF product is yet approved for wound healing, but research continues, including combinations of VEGF with other factors (since angiogenesis alone is not sufficient if other aspects are lacking) [22].

Beyond vascular regeneration, targeted stimulation of epithelial cell populations has also been explored through the use of keratinocyte-specific mitogens such as keratinocyte growth factor (KGF). KGF is produced by fibroblasts and specifically stimulates keratinocyte migration, proliferation, and differentiation. KGF-2 (repifermin) was tested in venous ulcers in early trials, and it was hypothesized to speed re-epithelialization. While a phase II trial in the early 2000s suggested some acceleration of healing, larger trials did not show significant benefit, and development was halted [18]. Nonetheless, recombinant KGF (palifermin) is successfully used to heal and protect oral mucosal lesions (in oncology), indicating its strong pro-epithelial effect; this concept might be revisited for skin in the future [23].

In contrast to epithelial or angiogenic factors, some biologics exert their wound-healing effects indirectly through modulation of the immune response. One such example is granulocyte colony-stimulating factor (G-CSF). Though not a classical wound healing growth factor, G-CSF (which mobilizes bone marrow neutrophils) has been used in infected or ischemic DFUs to improve outcomes [24]. Systemic G-CSF can increase circulating neutrophils and perhaps enhance bacterial clearance in wounds. Some RCTs in infected DFU showed reduced amputation rates with G-CSF treatment. In the recent network meta-analysis, G-CSF ranked highly for improving healing rates and reducing amputations in DFU [19]. Its mechanism is likely via improving infection control and increasing growth factor release indirectly through immune cells. G-CSF is not routinely used for wounds due to cost and systemic effects, but it exemplifies how modifying the wound’s cellular milieu can aid healing.

In summary, recombinant growth factors act on specific molecular targets: PDGF primarily on mesenchymal cells (fibroblasts, smooth muscle), EGF on epithelial cells, FGF on fibroblasts/endothelial cells, and VEGF on endothelial cells (Figure 2). By selecting a factor, therapy can be tailored to the wound’s needs. For example, a very dry, atrophic wound might benefit from PDGF or FGF to build granulation tissue, whereas a clean granular base with stalled edge epithelialization might benefit from EGF to encourage keratinocyte advance. One limitation of single-factor therapy is that wound healing requires a coordinated sequence of signals; providing only one factor may not fully correct the healing impairment, which could explain variable results in clinical trials [25]. Additionally, supraphysiologic doses of a single factor can have diminishing returns or unwanted effects (e.g., prolonged PDGF use has been associated with local tissue overgrowth or theoretical cancer risk, though analyses have not confirmed any significant cancer incidence at typical usage) [20]. Recombinant growth factors provide single-target, high-potency interventions that address specific cellular deficits in wound healing, complementing PRP’s broader biologic stimulation. Considered alongside the PRP pathways depicted in Figure 1, Figure 2 emphasizes that PDGF, EGF, bFGF, VEGF, and related agents ultimately converge on the same core processes, fibroblast recruitment, angiogenesis, and re-epithelialization, highlighting the complementary rather than redundant roles of PRP and recombinant growth factor therapies in cutaneous wound repair.

#### 3.1.3. Comparative Biological Activity and Synergies

PRP and recombinant growth factor therapies share the common goal of delivering growth-promoting signals to the wound, but they differ fundamentally in composition and mechanism of delivery (Table 1). PRP is a “cocktail” approach. It provides a milieu of multiple factors derived from the patient’s own blood, more closely mirroring the natural wound healing environment (albeit in concentrated form). This polyfactorial nature can be advantageous in chronic wounds that suffer from multiple deficits (e.g., poor angiogenesis and prolonged inflammation, and inadequate cell proliferation). PRP’s fibrin gel also physically fills dead space and serves as a scaffold for cell infiltration, which recombinant single factors lack [17]. On the other hand, recombinant therapies offer precision: a known quantity of a specific bioactive agent is delivered, which allows for more standardized dosing and the possibility of meeting regulatory approval (as in the case of becaplermin). They can target a particular bottleneck in the healing process. For instance, applying PDGF to jump-start granulation in a pale, non-progressing ulcer. However, single factors may not address all aspects needed for healing.

Notably, there is substantial overlap in the biological pathways influenced by PRP versus recombinant factors. For example, PRP contains PDGF, EGF, FGF, VEGF, etc., so using PRP will inherently deliver all those signals (though in patient-specific ratios), whereas using a recombinant PDGF will affect primarily PDGF-responsive pathways. It has been observed that PRP often leads to similar outcomes as targeted growth factor therapy in clinical trials. In a network meta-analysis of DFU treatments, PRP, EGF, and PDGF were all associated with improved healing outcomes compared to standard care and had overlapping confidence intervals for healing efficacy [2]. Interestingly, EGF and PRP were ranked highest for healing speed, while PDGF and PRP were top-ranked for wound area reduction [19]. This may suggest that a broad-spectrum approach (PRP) has the potential to be similarly effective as the strongest single-factor approach, perhaps due to PRP’s synergistic action on multiple cell types. Another consideration is safety and immunological response: PRP being autologous is inherently biocompatible and carries essentially no risk of immune reaction. Recombinant proteins are generally human-identical, but repeated use could theoretically cause antibody formation (though this has not been a major issue with becaplermin or EGF in studies to date). PRP does require a blood draw and processing for each use, which can be a logistical hurdle, whereas a tube of growth factor gel is readily available but at a high monetary cost.

Finally, an emerging area is combining approaches: e.g., incorporating recombinant growth factors into PRP or platelet-rich fibrin matrices to harness both the scaffold and the high-dose single factor [26]. Preclinical studies suggest that embedding exogenous EGF or bFGF in a platelet gel can prolong its availability and enhance wound healing synergistically [27,28]. Gene therapy approaches aim to have the wound itself produce growth factors continuously (for example, plasmid PDGF or adenovirus VEGF delivered to wound cells), which might offer a sustained stimulus beyond the relatively short half-life of topical proteins [29]. Hybrid strategies, such as these, pairing PRP with recombinant or gene-based delivery, represent the frontier of biologic wound care, seeking to combine PRP’s autologous safety with the reproducibility and potency of engineered factors.

In summary, PRP offers a multi-pronged, autologous tactic, whereas recombinant growth factors provide targeted, standardized interventions. Table 1 outlines key differences and similarities between the two strategies in the context of wound healing.

**Table 1:** Summary of key differences between platelet-rich plasma (PRP) and recombinant growth factor therapy in cutaneous wound healing.

Feature	Platelet-Rich Plasma (PRP)	Recombinant Growth Factor Therapy
Composition	Autologous platelet concentrate delivering a cocktail of growth factors, cytokines, fibrin, and ± leukocytes.	Single bioengineered growth factor (e.g., PDGF-BB) at a defined concentration.
Mechanism of Action	Releases multiple factors spanning all healing phases; fibrin clot doubles as a cellular scaffold.	Triggers the specific receptor pathway of that factor, targeting one key phase of repair.
Delivery/Form	Mixed bedside from patient blood; applied as clot/gel or peri-wound injection, patient-specific each time.	Factory-made gel/solution with fixed dose; applied or injected on a set schedule.
Advantages	Autologous, minimally immunogenic; broad biologic coverage and scaffold effect; useful across diverse wound types.	High, reproducible potency; standardized dosing simplifies trials; usable when blood draw is impractical.
Limitations	Content varies by patient and kit; needs centrifuge, immediate use, and often repeat applications.	Targets only one pathway; expensive, cold-chain sensitive, and carries factor-specific adverse-event warnings.

### 3.2. Clinical Applications Across Wound Types

**Figure 3:**
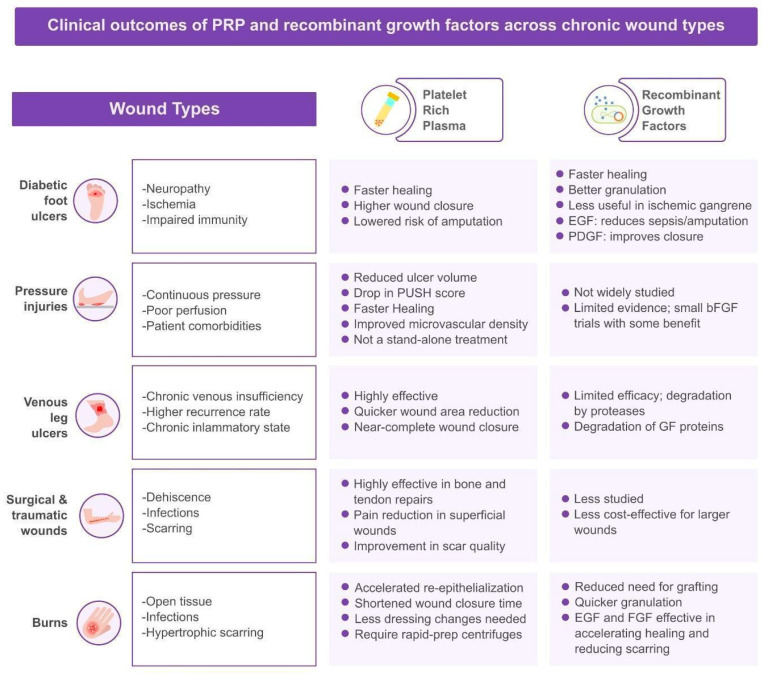
Summarized comparison of the clinical outcomes of PRP and recombinant growth factors across chronic wound types.

Emerging evidence suggests that PRP and growth factor therapies may benefit a spectrum of wound types (Figure 3). Here, we review their clinical applications and outcomes in major wound categories, highlighting comparative effectiveness where data exist.

To help readers interpret the clinical evidence, we summarize in Table 2 the key randomized controlled trials and meta-analyses of PRP and recombinant growth factor therapies across the major wound types. Research findings demonstrate that PRP, used as an adjunctive therapy, leads to better wound closure outcomes and faster healing times for diabetic foot, venous leg, and pressure ulcers compared with standard treatment protocols. Topical and injectable growth factors, including PDGF-BB, EGF, and bFGF, also improve epithelialization, reduce ulcer area, and shorten healing time in chronic ulcers and burns. Together, these data form the basis for our conclusions regarding the role of PRP and recombinant growth factors as adjunctive treatments in cutaneous wound healing. The wound-specific subsections that follow provide a narrative interpretation of these findings in the context of routine clinical practice.

#### 3.2.1. Diabetic Foot Ulcers

Chronic foot ulcers in diabetic patients are notoriously challenging to heal due to neuropathy, ischemia, and impaired immune function. Both PRP and recombinant growth factors have been extensively studied in diabetic foot ulcers (DFUs), with the goal of promoting closure and preventing amputations. New studies continuously show that adding PRP to regular care helps diabetic foot ulcers close faster and more completely. In meta-analyses of RCTs, patients treated with autologous PRP (in addition to good wound care) had markedly higher complete-healing rates than those receiving standard care alone [5]. For example, a 2022 systematic review (29 RCTs, >2000 wounds) found PRP was associated with an approximate five-fold increase in the odds of full wound closure (OR ≈ 5.3) compared with controls [5]. PRP-treated DFUs also showed larger reductions in wound area and generally healed faster. Subgroup analyses indicate that the benefit extends to both neuropathic and neuro-ischemic ulcers, though results may be strongest in purely neuropathic lesions (which face fewer arterial-supply issues) [5]. A 2023 network meta-analysis ranked PRP among the leading DFU treatments: patients were about 3.4 times more likely to reach full closure than with standard care [2]. Patients who received PRP needed fewer major amputations, probably because their wounds healed more quickly and stayed infection-free [19]. PRP is generally safe for people with diabetes. Trials have not seen more side effects or deaths, and some even note fewer infections, perhaps thanks to the white cells and natural antimicrobial proteins in the platelet mix [5,19]. Clinically, PRP can be applied as a topical gel dressing or injected around/into the ulcer; some protocols do both, injecting the edges and covering the surface with gel. Taken together, the evidence supports PRP as a useful add-on for stubborn DFUs, with multiple trials and reviews reporting better healing [2,5]. Older guidelines were cautious, but the growing pile of positive studies, some showing lower costs from fewer amputations and hospital stays, now makes PRP a strong option when standard care is not working.

Turning from autologous biologics to engineered single-factor products, recombinant growth factors provide another therapeutic avenue for DFUs. Diabetic foot ulcers were the first wounds to receive such an agent. Becaplermin (recombinant PDGF-BB) gained FDA approval in 1997 after registration trials showed higher healing rates when it was added to good wound care [30]. Contemporary evidence keeps bolstering PDGF’s value: a 2025 network meta-analysis ranked it among the top performers for complete closure in DFUs [19]. Likewise, a pooled analysis of four RCTs reported that daily PDGF gel roughly doubled the likelihood of healing by 20 weeks [2]. The gel works best in superficial, well-perfused ulcers and is far less useful in deep abscesses or ischemic gangrene [31]. In practice, it is applied once daily for up to 20 weeks or until closure. Cost can be a concern, yet a 2016 cost-utility model found becaplermin to be economically dominant over standard care alone because faster healing reduced downstream expenses [32]. Safety fears emerged in 2008, when a retrospective signal suggested more malignancies after prolonged, high-tube exposure, prompting an FDA boxed warning; however, the agency removed that warning in 2018 after larger follow-up analyses showed no excess mortality [30], and a matched-cohort study likewise found no significant increase in cancer incidence with routine, short-term use [33]. Overall, current data indicate that the limb-salvage benefit of short-course becaplermin outweighs these earlier theoretical risks, particularly in appropriately selected, well-perfused DFUs [17].

Beyond PDGF, attention has shifted to recombinant epidermal growth factor (EGF). Cuba first deployed intralesional EGF (Heberprot-P) for advanced DFUs, injecting the peptide directly into the wound bed two or three times a week; real-world cohorts have reported ≥50% granulation by eight weeks and a marked fall in major amputations [34]. Controlled data back this up: a phase III multicenter RCT of spray-applied rhEGF more than doubled the complete-healing rate versus placebo at 12 weeks [35], while a 2020 pooled analysis confirmed that EGF accelerates closure and limb preservation [36]. The most recent network meta-analysis covering 112 trials ranked EGF highest for boosting DFU healing probability among all tested growth factors [19]. Although currently licensed in only a handful of countries (Cuba, Vietnam, several Middle Eastern states), uptake is growing wherever injection infrastructure and funding allow.

Similar principles apply to fibroblast growth factor (FGF), particularly the basic isoform (bFGF) used topically in East Asia. A Chinese randomized study of 199 patients showed that bFGF spray produced faster granulation and a larger percentage reduction in ulcer area over eight weeks than standard care [37]; these findings are echoed in a 2021 umbrella review of growth factor therapy [38].

At the vascular end of the spectrum, VEGF gene therapy has been tried in “no-option” ischemic DFUs to spur collateral vessel growth. A 24-patient Polish study that sequentially injected VEGF/HGF and ANG-1 plasmids noted improved ankle–brachial indices and roughly a 50% drop in major amputations at three months, though the small sample underlines the approach’s inconsistency [39]. A 2023 systematic review similarly found that three of four clinical studies showed faster healing with VEGF, but overall certainty remains low [40]. Finally, granulocyte-colony stimulating factor (G-CSF) is sometimes added when infection complicates a DFU. By boosting neutrophil activity, it can tilt the balance in severe sepsis; a 2021 evidence review concluded that systemic G-CSF reduced major amputation risk even though it did not significantly hasten ulcer closure [41].

When these modalities are compared head-to-head, a few themes emerge. Direct comparisons are few, but mixed-treatment comparisons (NMAs) give some insight. As mentioned, EGF, PRP, and PDGF all significantly improved healing rates vs. standard care in DFU [2]. EGF had the highest point estimate for efficacy, followed by PRP, then PDGF [2]. PRP and EGF also showed the greatest shortening of healing time (likely by accelerating granulation and re-epithelialization) [19]. PDGF was especially good at reducing ulcer area (consistent with its effect on granulation tissue) [19]. PRP stood out in safety. It was associated with fewer adverse events overall, and interestingly, the lowest amputation rates among the treatments analyzed [19]. This could reflect PRP’s polyfactorial influence on angiogenesis, immunity, and infection control, rather than single-pathway stimulation. In practical terms, a clinician might choose PRP for a patient who can have blood drawn and where a broad stimulation is desired, whereas PDGF gel might be preferred if resources for PRP processing are not available (but the patient or system can afford the medication). EGF could be reserved for the most refractory cases or where intralesional injection infrastructure exists. Combination therapy, such as alternating PRP and EGF applications, has not been widely studied; however, it could offer additive benefits by combining autologous and engineered mechanisms. Until more data emerge, practitioners must rely on available resources and patient specifics, with the comforting knowledge that all these approaches tend to improve outcomes when used appropriately alongside standard wound care (offloading, debridement, infection control, and perfusion optimization).

Both PRP and recombinant factors, such as PDGF and EGF, significantly enhance healing in diabetic foot ulcers. However, PRP provides broader biologic coverage, while PDGF offers standardized dosing and proven regulatory approval.

#### 3.2.2. Pressure Injuries

Pressure ulcers (PUs), especially stage III/IV lesions over bony prominences in bedridden patients, have prolonged healing courses due to continuous pressure, often poor perfusion, and patient comorbidities [42]. Advanced therapies like PRP and growth factors have been explored to jump-start healing in these wounds as well [43].

The evidence base, while smaller than for DFUs, indicates a benefit of PRP in treating pressure injuries. A 2024 meta-analysis of 9 RCTs involving 523 pressure ulcers reported that adjunctive PRP significantly improved healing outcomes [8]. The pooled healing rate in the PRP-treated group was significantly higher (odds ratio ~3.4) than in controls receiving standard care [8]. PRP also led to greater reductions in wound size and depth, as reflected in improved Pressure Ulcer Scale for Healing (PUSH) scores (PRP patients had a significantly larger drop in PUSH score, meaning better healing progression) [8,44]. Importantly, these benefits were achieved without an increase in complications. Infection rates, pain levels, and adverse events were similar between PRP and control groups [45]. Clinicians have applied PRP to pressure ulcers either by injecting it into the wound edges or by soaking a dressing in PRP gel and filling the ulcer cavity [44]. Case series illustrate scenarios like a sacral ulcer with exposed bone that, after serial PRP applications, developed healthy granulation tissue covering the bone and eventually closed [46]. Mechanistically, PRP likely helps overcome the chronic inflammatory environment of PUs by delivering needed growth factors to ischemic tissues compressed by pressure. The angiogenic factors in PRP (VEGF, FGF) may improve microvascular density in the wound bed, while PDGF and EGF stimulate the sluggish granulation and re-epithelialization often seen in PUs [47].

It is worth noting that pressure ulcers often coexist with poor nutrition and perfusion; thus, addressing those systemic issues remains paramount [48]. PRP is an adjunct, not a standalone cure-all. In the meta-analysis, PRP did not significantly reduce ulcer volume in every case, and in some studies, the difference in complete healing time was not statistically significant [8]. This suggests that while PRP can significantly tilt the odds in favor of healing, standard measures (offloading pressure, surgical flap closure if indicated, protein supplementation, etc.) are still essential. Nevertheless, given the relatively low risk and the positive evidence, PRP therapy is increasingly considered for Stage III/IV pressure ulcers that are not improving with conventional treatment.

There is less clinical trial data for growth factor gels or injections in pressure sores. One reason is that after becaplermin’s success in DFUs, efforts to test it in pressure ulcers were limited (possibly due to different wound biology and the expense of large trials) [49]. A few small studies have examined topical PDGF or FGF on pressure ulcers, with mixed results [21]. An older RCT on PDGF gel in pressure ulcers did not show as clear a benefit as in DFUs, possibly because pressure ulcers often have significant tissue necrosis and require aggressive debridement beyond what a growth factor alone can address. Basic FGF spray has been used in Japan for pressure ulcers with some reported success in case series (faster granulation tissue formation). In one comparative study, bFGF-treated pressure ulcers achieved >90% granulation coverage more quickly than controls [2]. Another avenue is GM-CSF (granulocyte–macrophage colony-stimulating factor) applied topically; a small trial suggested that GM-CSF can improve healing in paraplegic patients’ pressure sores by boosting local immune cell activity [50], but this is not widely adopted.

Overall, no recombinant factor has an approved indication for pressure ulcers at this time. The heterogeneity of pressure ulcers (location, size, patient condition) makes standardized trials difficult. However, clinicians sometimes use becaplermin off-label for pressure ulcers that are clean but granulation-deficient. If used, it should be in a wound free of necrosis and infection, and combined with pressure relief and appropriate wound bed preparation. The cost-effectiveness in this population is unclear, as many pressure ulcers occur in older patients or those with limited life expectancy, where the impetus is on comfort and basic care [51]. PRP might have an edge here because of its safety profile and ability to be prepared bedside in long-term care settings [45].

In summary, for pressure ulcers, PRP has the most supportive evidence to date, showing improved healing rates and wound contraction [8]. Growth factor therapies are less established, though they may still be considered on a case-by-case basis. Future RCTs focusing on specific subgroups (e.g., young spinal cord injury patients with stage IV sacral ulcers) could further clarify the role of these biologics in pressure ulcer care. Given the burdensome nature of pressure injuries, any intervention that safely accelerates healing is of great clinical value.

#### 3.2.3. Venous Leg Ulcers

Venous leg ulcers (VLUs) are ulcers typically on the gaiter area of the lower legs, caused by chronic venous insufficiency. Standard therapy includes compression bandaging and wound care, but healing can be slow, and recurrence is common. Both PRP and growth factors have been investigated to enhance VLU healing. Evidence suggests PRP may be particularly effective in venous ulcers. A systematic review stratifying wound trials by etiology found that all studies on venous ulcers reported improved healing with PRP therapy compared to controls [25]. Notably, the analysis suggested that the signal for PRP’s efficacy appeared strongest in venous ulcers among the wound types included. For instance, PRP-treated VLU patients showed faster reduction in ulcer area and higher rates of complete closure within the study periods. One reason for this may be that venous ulcers generally retain adequate arterial perfusion, allowing platelets and cytokines in PRP to function optimally once the chronic inflammatory environment is modulated. Also, many venous ulcers are stuck in a chronic inflammatory state (with heavy exudate and elevated matrix metalloproteinases); PRP, with its anti-inflammatory cytokines and protease inhibitors, might help modulate that environment towards healing [17,52].

A notable RCT in 2018 on refractory VLUs showed that weekly PRP injections plus compression led to significantly greater wound-area reduction at 12 weeks than compression alone. Some chronic ulcers that had not healed in months achieved near-complete closure with the addition of PRP. Another study used PRP gel applied under the compression bandage with dressing changes; the PRP group had a higher proportion of wounds healed by 16 weeks. PRP is typically used after adequate debridement; the platelet gel can be spread on the ulcer surface (often in combination with a secondary dressing like foam to keep it in place under compression). Patients have tolerated this well, with no difference in pain or infection noted between PRP and controls in meta-analyses [25]. In fact, one meta-analysis found venous ulcers responded better to PRP than diabetic ulcers did, possibly due to differences in wound chronicity and PRP application methods (in that analysis, more venous ulcer studies used injected PRP, which was associated with better outcomes) [5].

The track record for single growth factors in VLUs is less encouraging. PDGF was tried in the 1990s for VLUs without much success, which was somewhat surprising since one would expect PDGF to help granulation similar to DFUs. It is hypothesized that the high protease activity in chronic venous ulcer fluid might degrade single factors before they can act. A Cochrane review on growth factors for VLUs (2022) found that, when pooling results, growth factor treatments (various kinds, including PDGF, EGF, and others) modestly increased the chance of complete healing compared to standard care [53]. However, the quality of evidence was low, and results were inconsistent across trials [54]. One of the largest trials in venous ulcers was with repifermin (KGF-2), which ultimately showed no significant benefit in healing rates at 20 weeks compared to the placebo, despite earlier dose-finding studies suggesting some effect [53]. Basic FGF has shown promise in some smaller studies: e.g., a Japanese study reported that VLUs treated with bFGF had better early healing indices. EGF topical spray was tried in a small trial for VLUs in India, with some improvement noted, but more evidence is needed [21].

Given that compression therapy addresses the root cause (venous hypertension) and is very effective when adhered to, the incremental benefit of expensive biologics in VLUs must be weighed. For a stubborn venous ulcer that is not closing despite good compression and wound care, some clinicians have used becaplermin gel off-label. There are case reports of chronic VLUs finally healing after patients were put on PDGF gel for 8–12 weeks, but RCT-level proof is sparse. The aforementioned network meta-analysis (Tian et al., 2025) indirectly suggests that VEGF might be beneficial (since in DFUs, VEGF ranked second for healing rate behind EGF), and venous ulcers likely also benefit from angiogenesis; but no formal VEGF trials in VLUs have been published to our knowledge [19].

PRP appears to be a strong option for venous ulcers, with systematic reviews concluding that PRP therapy improves VLU healing rates [25]. It can be considered when conventional therapy alone is not leading to progress. Growth factors like PDGF or FGF might offer some benefit, but they are not standard for VLUs, and insurance often will not cover them for this indication. Given PRP’s autologous nature and relative ease of use in an outpatient setting, it may have a more practical role in VLU clinics. Importantly, any adjunct must be combined with compression; biologics without compression in VLUs are unlikely to succeed, because the underlying venous pressure must be controlled for healing to occur.

#### 3.2.4. Surgical and Traumatic Wounds

Acute surgical wounds (e.g., incisions, donor sites) and traumatic wounds (e.g., lacerations, degloving injuries) usually heal if well approximated or grafted, but there is interest in PRP and growth factors to accelerate healing and improve scar quality in these contexts. Additionally, some surgical wounds, like sternotomy wounds or large excisional wounds, can have complicated healing (dehiscence, infection) where biologic adjuncts might help.

PRP has been used in various surgical specialties to promote tissue repair. Its benefits in bone grafting and tendon repairs are well-documented in orthopedics. For skin wound healing specifically, evidence is mixed. A recent systematic review in plastic surgery applications concluded that PRP’s role as a routine adjuvant is not yet justified for skin grafting or burn surgery [52]. In that analysis of multiple RCTs, PRP did not significantly improve skin-graft take percentage overall. However, the same review found a clear benefit for PRP in donor-site healing. Pooled data from trials showed that split-thickness skin-graft donor areas healed faster (by ~5.5 days on average) when PRP was applied, compared to standard dressings [52]. Patients also reported less pain with PRP-treated donor sites, possibly because PRP’s fibrin prevented the dressings from sticking and reduced local inflammation. These findings suggest that PRP may not always alter graft take, but it can significantly accelerate re-epithelialization and improve patient comfort in donor and secondary healing sites.

For acute surgical incisions, some surgeons have injected PRP along high-risk closures (e.g., after pilonidal sinus excision or abdominoplasty) to reduce wound complications. There are limited RCT data here; small studies indicate PRP may reduce wound dehiscence rates and improve scar quality (owing to more organized collagen deposition). One RCT on cesarean-section incisions found no difference in primary healing but noted slightly better scar pliability at 6 weeks in the PRP group. In chronic non-healing surgical wounds (like a dehisced sternal wound), PRP has been successfully used to promote granulation and avoid the need for reoperation, as case reports indicate [52].

Traumatic wounds with significant tissue loss or contamination can benefit from PRP’s hemostatic and regenerative properties. PRP gel can be applied to clean traumatic wounds after debridement to encourage granulation. In wounds with exposed structures (like tendons or bone) that are not immediately flap-covered, PRP has been shown to stimulate granulation tissue covering the exposed structures, thus converting them to a cleaner wound bed for later closure [55]. A preliminary study of 12 patients with “refractory” wounds with exposed tendons (from trauma, burns, or complications) treated with PRP injections reported that all wounds achieved sufficient granulation to cover the tendon and went on to heal, with improved scar scores and no infections [55]. This highlights PRP’s potential in complex wound management, essentially as a bridge therapy to facilitate subsequent closure by secondary intention or delayed grafting.

Recombinant growth factors in surgical or traumatic wounds have not become mainstream, but a few niche uses exist. For example, recombinant PDGF has been tried to reduce wound-healing time in acute surgical wounds in patients prone to poor healing (like those on steroids or the elderly). Some surgeons apply becaplermin gel along a laparotomy incision in high-risk patients as prophylaxis for wound-healing complications; however, no large trial validates this practice. FGFs have potential in acute wounds; one trial found applying bFGF to palatal-mucosa donor sites (after gum surgery) significantly hastened mucosal healing vs. placebo [56]. This suggests growth factors could improve mucosal or cutaneous healing in acute settings, but more research is needed. For traumatic wounds, particularly those that are partial-thickness (abrasions or second-degree burns), topical EGF or FGF could theoretically speed re-epithelialization; small studies in burn units show earlier epithelial cover by a day or two with growth factor sprays [57]. A unique scenario is chronic surgical wounds (like a non-healing post-operative wound). PDGF gel has been used off-label in such cases. For instance, a chronic non-union of a surgical incision or a sinus tract might respond to growth factor stimulation [58]. G-CSF has even been used in infected sternotomy wounds to aid clearance [19]. These are rescue situations rather than routine.

In clean, acute surgical wounds that are expected to heal primarily, the incremental benefit of PRP or growth factors is likely small, and trials indeed show little difference in uncomplicated cases [52]. The role for these therapies in surgical/traumatic wound care is more prominent in compromised or complicated situations: e.g., large wounds, areas of marginal perfusion, or when normal healing is impaired. PRP shines in promoting secondary healing (donor sites, granulating wounds), whereas recombinant factors have not proven cost-effective for routine use. Ongoing studies (one referenced in 2025 is evaluating PRP in burn-patient skin grafts) will further clarify if certain acute-wound scenarios can benefit significantly [52]. Until then, usage in this domain is at clinicians’ discretion, balancing evidence and considering that PRP application in a serious wound likely will not hurt and might help, whereas growth factor products must justify their cost.

#### 3.2.5. Burns

Burn wound management has increasingly turned to biologic adjuncts that can shorten the hazardous window of open tissue and thereby limit infection, scarring, and hospital days. Two strategies dominate the literature of the past decade: autologous platelet-rich plasma or fibrin (PRP/PRF) and pharmaceutical-grade recombinant growth factors. Although they work through overlapping molecular cascades, their clinical profiles, logistical demands, and evidentiary support differ in meaningful ways. A 2024 systematic review by Manasyan et al. pooled 14 studies involving 781 patients and concluded that PRP/PRF was associated with accelerated re-epithelialization of partial-thickness burns, sped donor-site closure, and improved some reports of skin-graft take, all without increasing adverse events [57]. In comparison, the 2023 plastic-surgery-oriented review by Knightly et al. judged the effect of PRP on graft acceptance to be equivocal, attributing the discrepancy to heterogeneous protocols and outcome definitions [52]. Even so, both reviews agreed that PRP shortened time to wound closure, a clinically decisive endpoint because every lost day of re-epithelialization multiplies infection risk. These findings echo earlier single-center observations that PRF membranes, laid either over donor sites or directly on debrided deep dermal burns, acted as biologic dressings that released growth factors over days and visibly accelerated epithelial coverage while posing no infection or hypertrophic scar penalty [17].

Quantitative syntheses strengthen that qualitative picture. A 2021 meta-analysis of eight trials (449 patients) showed that PRP shaved a mean of 3.5 days from burn closure compared with standard care and did so with no difference in infection rate or graft integration [59]. Likewise, an RCT that sprayed PRP beneath split-thickness grafts on excised deep burns detected no early advantage in graft adherence or epithelialization by day 7 and found scars comparable at one year [60]. Yet, when nine more recent RCTs were added to the evidence stream, a 2025 meta-analysis involving 413 participants recalculated the benefit: burns treated with adjunctive PRP healed an average of 6.7 days faster, dressing changes fell, and the odds ratio for infection plummeted to about 0.18, again without extra pain or graft-take benefit [61]. The divergence among pooled estimates probably reflects burn-depth heterogeneity and the timing of PRP delivery; nonetheless, a week-long acceleration is clinically meaningful, especially when it halves the number of dressing changes and, by extension, resource utilization. Such a reduction in closure time—roughly a week—is clinically meaningful, resulting in decreased hospital stays, antibiotic use, and overall cost of care. Practical downsides, such as extra venipunctures for large total-body-surface-area injuries and operating-room minutes to spin fresh PRP, remain modest relative to the gains.

Emerging evidence that includes next-generation PRF appears even more favorable. Manasyan et al.’s broader inclusion criteria captured PRF’s sustained-release matrix effect and tilted the weight of the literature toward recommending platelet concentrates as “helpful adjuncts” for both superficial burns and graft donor sites [57]. Although Knightly et al. urged caution about high study heterogeneity, they did concede shorter healing times and reiterated that no study has shown PRP to worsen outcomes [52]. That safety profile is critical: unlike exogenous cytokines, autologous PRP is nearly devoid of immunologic risk, and no trial has recorded higher infection, abnormal scarring, or carcinogenic signals. Logistics, rather than biologic hazard, therefore limit adoption; burn centers without rapid-prep centrifuges may find bedside PRP impractical in mass-casualty scenarios, whereas high-resource units now spray PRP on excised beds or sandwich it beneath meshed grafts as routine.

The use of second-generation platelet concentrates like platelet-rich fibrin (PRF) in burn and reconstructive surgery has increased, but PRP remains more suitable for various cutaneous applications because of its practical features [52,57]. Its liquid consistency enables practitioners to utilize injectable and sprayable application methods, allowing healthcare providers to infiltrate complex wounds or use it during surgical procedures like skin grafting and negative-pressure therapy [52]. Furthermore, the majority of research studies regarding platelet treatment for burns and chronic ulcers have been conducted with PRP, making it the leading evidence-based option currently [5,52,57]. The pre-formed fibrin matrix in PRF does offer extended growth factor delivery, which benefits donor sites and specific burn injuries, yet its delivery methods are limited and supported by fewer high-quality studies for chronic wound treatment [57]. Thus, PRP and PRF operate as distinct treatment options where their physical characteristics suit different applications in burn and wound care [52,57].

Recombinant growth factors attack the same bottleneck, slow epithelial resurfacing, but through single-molecule precision. A sweeping 2022 meta-analysis of 281 studies, many in East Asian burn cohorts, demonstrated that topical epidermal growth factor (EGF), basic fibroblast growth factor (bFGF), or granulocyte–macrophage colony-stimulating factor (GM-CSF) reduced healing time in superficial burns by roughly three days and in deeper second-degree burns by more than five, while simultaneously lowering scar-severity scores and adverse-event rates [62]. Trial-level data illustrate those averages: Lee et al. reported that a low-dose rh-EGF ointment closed second-degree burns in about 12–13 days (standard care typically exceeds two weeks), and adding EGF to a biologic skin substitute dropped mean closure to nine days [63]. In Japan, bFGF (trafermin) has regulatory approval after RCTs showed quicker granulation and reduced need for grafting; treated wounds often exhibited more elastic scars on follow-up. GM-CSF topical therapy remains investigational, but pooled analyses still reveal faster closure with no systemic toxicity. The mechanistic allure is obvious. EGF drives keratinocyte proliferation, bFGF jump-starts angiogenesis, and GM-CSF modulates early immune influx. Yet, the narrow target range of each molecule means they correct only specific deficits. Their practicality also depends on access and cost; while recombinant agents offer standardized dosing, their expense and cold-chain needs limit widespread use outside high-resource settings.

Rather than forcing a binary choice, investigators now test synergistic regimens: loading rh-EGF or bFGF into PRF gels prolongs cytokine availability and closes animal burns faster than either modality alone, and one registered 2025 RCT will irrigate excised human burns with PRP in theater and then maintain outpatient donor sites with daily EGF spray [57]. Conceptually, PRP supplies the scaffold and a baseline multipronged stimulus, while a recombinant factor boosts whichever signaling axis remains rate-limiting, such as keratinocyte mitogenesis for EGF, angiogenesis for bFGF, or immune modulation for GM-CSF. Such layering may finally nudge the stubborn endpoints, graft adherence, and ultimately scar pliability, factors that have resisted improvement in single-agent trials. Future iterations of combination therapy may integrate gene-encoded growth factors or bioprinted PRF matrices for controlled, phase-specific cytokine release, aligning biologic activity with the wound’s healing stage.

In summary, contemporary evidence demonstrates that both autologous platelet concentrates and recombinant growth factors meaningfully accelerate closure of partial-thickness burns, with PRP/PRF also curtailing infection odds and recombinant agents showing marginally better scar profiles in some series. Neither intervention materially changes graft adherence across studies to date, but each shortens hospital stays, dressing frequency, and overall nursing burden. Their safety records remain robust: no increase in systemic toxicity, aberrant scarring, or neoplasm has been documented. When resources permit, integrating PRP or PRF intra-operatively with postoperative recombinant-factor dressings may combine autologous safety with molecular precision, optimizing both speed and quality of burn healing.

**Table 2:** Key Clinical studies and meta-analyses of PRP and recombinant growth factors in wound healing.

Wound Type	Intervention	Study Type	Key Findings	References
Diabetic Foot Ulcer (DFU)	PRP (Autologous)	Systematic Review & Meta-Analysis (2022)	In chronic ulcers (including DFU), PRP increased complete closure (overall OR ≈ 5.3; DFU subgroup OR ≈ 2.3) and significantly reduced residual wound area versus standard care.	[5]
	PDGF-BB (Becaplermin)	Network Meta-Analysis (2025)	PDGF-BB improved DFU healing compared with standard care and ranked among the most effective agents for reducing ulcer area in the network analysis.	[19]
	rh-EGF (Injection/Spray)	Phase III RCT/Pooled Analysis	Topical rh-EGF spray increased complete healing and shortened time to closure versus placebo; intralesional rh-EGF achieved ≥50% granulation by ~8 weeks and high limb-salvage rates in advanced DFU.	[34,35,36]
Pressure Injury	PRP	Meta-Analysis (2024)	PRP increased healing rates (OR ≈ 3.4), reduced ulcer size and PUSH scores, and shortened healing time versus standard dressings, without higher adverse-event rates.	[8,44]
	bFGF	Comparative Study/NMA	Topical bFGF accelerated granulation and re-epithelialization in chronic ulcers and burns; small studies suggest benefit in pressure ulcers, but evidence remains limited.	[2,21]
Venous Leg Ulcer (VLU)	PRP	Systematic Review/RCT	Adjunct PRP with compression produced greater ulcer-area reduction and higher complete-healing rates at ~12 weeks than compression therapy alone across multiple RCTs.	[25]
Burns	PRP/PRF	Systematic Review & Meta-Analyses (2024/2025)	PRP/PRF shortened re-epithelialization of burns and donor sites and improved graft or donor-site quality; some trials also reported fewer dressing changes and infections.	[57,59,61]
	rh-EGF/bFGF	Meta-Analysis (2022)	Topical EGF/bFGF reduced healing time in superficial burns by ~3 days and in deeper burns by >5 days versus conventional dressings and improved scar outcomes.	[62]

### 3.3. Limitations and Practical Considerations for PRP and Recombinant Growth Factors

Although the outcomes of the studies are encouraging, the extensive use of platelet-rich plasma (PRP) and recombinant growth factor interventions in treating skin wounds is still limited by several crucial drawbacks. To begin with, PRP preparations differ from each other. The distribution of platelets, leukocyte components, the activation method, and the application frequency vary considerably among different protocols and commercial systems, which makes it almost impossible to either correlate different studies or define the “best” formulation [9,13]. This heterogeneity is likely the reason for the inconsistent results across trials and makes regulatory approval and reimbursement more complicated [5,25].

Second, PRP and growth factor products are burdened with logistical and cost issues. The preparation of autologous PRP necessitates the presence of specialized healthcare personnel, specific centrifugation kits, and point-of-care processing time, all of which may not be obtainable in low-resource health facilities or high-volume outpatient clinics [57]. On the other hand, recombinant growth factors, which are standardized, oftentimes necessitate repeated applications over a period of weeks and are significantly more costly than the usual dressings, resulting in limited access in a number of health systems [32,51].

Third, the field is still dominated by small studies. Although randomized studies and meta-analyses are available, they are sometimes lacking in sample size, follow-up, and enumeration of endpoints [2,5]. Exceptionally efficient, well-designed, and multicenter trials carried out on PRP, specific growth factors, and standard care in a head-to-head manner are conspicuously lacking. For this reason, the problem of inability to delineate the appropriate patients needing the therapy the most and of obtaining clinical guidelines based on derived evidence are difficulties that are experienced [19].

Last but not least, patient challenges and wound details have an effect on biologic treatments. For example, it has been cited that severe ischemia, uncontrolled infection, malnutrition, and poor glycemic control might negatively affect the healing of the wound despite the specific product introduced [64]. On the other hand, for the case of recombinant growth factors, there is the theoretical assumption that they could stimulate abnormal tumor-like changes or aberrant scarring with prolonged exposure, which is, of necessity, a caution in patient selection, especially in the presence of a history of malignancy or dysplastic skin changes [30,33]. Hence, these limitations together signify that PRP and recombinant growth factors are not cures but should be seen as additional medications to meticulous modern standard wound care.

Finally, the inclusion of heterogeneous study designs, ranging from preclinical models to network meta-analyses, precludes direct statistical comparison between all therapeutic agents. Consequently, the comparative efficacy rankings discussed here reflect the findings of specific component studies rather than a unified systematic synthesis.

## 4. Future Directions

Large, head-to-head clinical trials are still the missing piece in the PRP and growth factor puzzle. Although smaller studies hint at real benefits, their designs vary so widely that it is hard to compare results across centers or even replicate a protocol two doors down the hall. Multicenter, randomized projects, preferably with harmonized platelet counts, application schedules, and clear outcome definitions, would give the field a much sturdier foundation [20]. A second, quieter problem is the tendency to describe a PRP recipe without reporting the actual growth factor payload. Until groups agree to quantify these biologic “doses”, we will keep arguing over why two superficially similar trials diverged so sharply in their conclusions [65]. Better documentation would also let investigators tease out which patient traits, such as poor glycemic control, smoking, obesity, or something else entirely, blunt the response to treatment [64].

There is growing excitement around pairing biologic therapies with the best of modern wound care. Think of PRP layered beneath a negative-pressure dressing or woven into a tissue-engineered scaffold: early reports suggest the combination speeds granulation and closure more than either strategy on its own [66]. The same logic is driving trials that add autologous PRP to split-thickness skin grafting, especially in burns, where every day of open tissue invites infection [67]. If these studies confirm higher graft take or fewer dressing changes, surgeons may soon view biologic adjuncts the way they now view antibiotic prophylaxis: helpful, inexpensive insurance against common complications.

The toughest tests, however, still lie with chronic ulcers: diabetic foot wounds, venous leg ulcers, pressure sores, and similarly stubborn lesions confound clinicians and strain healthcare budgets worldwide. Future protocols that integrate PRP or recombinant factors must therefore look beyond simple closure times. Investigators need to ask whether these treatments lower infection rates, reduce rehospitalizations, or ultimately keep a patient’s limb intact [66]. Cost-effectiveness and quality-of-life metrics should move from the appendix to center stage, especially as off-the-shelf products such as platelet-derived exosomes and allogeneic PRP blends inch closer to market release [64]. If the next wave of studies can show that biologic dressings pay for themselves by shortening admissions or preventing amputations, guidelines will have a compelling reason to elevate these therapies from promising adjuncts to a routine standard of care.

## 5. Conclusions

The present review delineates how platelet-rich plasma (PRP) and recombinant growth factor products collectively broaden the therapeutic armamentarium for difficult-to-heal cutaneous wounds. PRP supplies an autologous, polyfactorial milieu and a provisional fibrin scaffold that can restart stalled repair in diabetic, venous, pressure, and burn injuries, whereas single-factor biologics such as PDGF-BB, EGF, and bFGF deliver high-potency, target-specific stimuli that address discrete cellular bottlenecks. When used in conjunction with meticulous debridement, off-loading, infection control, and optimization of tissue perfusion, both approaches have repeatedly shortened re-epithelialization timelines without introducing notable safety concerns. This suggests that biologic adjuncts are ready for wider clinical uptake where resources and expertise permit.

Even so, the evidence base remains fragmented: variations in PRP processing, recombinant-factor dosing, outcome selection, and follow-up duration limit meaningful comparisons and obscure cost-effectiveness. Multicenter trials that adopt harmonized preparation protocols, standardized cytokine assays, and patient-centered endpoints, such as limb preservation, quality-of-life metrics, and nursing workload, are needed to clarify true clinical value. Parallel investigations should rigorously test combination strategies (e.g., platelet matrices paired with recombinant cytokine depots, negative-pressure therapy, or bio-printed scaffolds) to identify regimens with genuine synergistic benefit. Success along these lines would enable PRP and growth factor therapeutics to evolve from intriguing adjuncts into routinely deployed, evidence-based tools in modern regenerative wound care.

## Data Availability

All data reported are available in the PubMed and Google Scholar databases.

## Abbreviations

The following abbreviations are used in this manuscript:

PRP	Platelet-Rich Plasma
PDGF-BB	Becaplermin
DFU	Diabetic Foot Ulcer
PDGF	Platelet-Derived Growth Factor
TGF-β	Transforming Growth Factor-β
EGF	Epidermal Growth Factor
VEGF	Vascular Endothelial Growth Factor
FGF	Fibroblast Growth Factor
IGF-1	Insulin-like Growth Factor
RCT	Randomized Controlled Trial
HER1	Human Epidermal Growth Factor Receptor 1
bFGF	Basic Fibroblast Growth Factor
KGF	Keratinocyte Growth Factor
G-CSF	Granulocyte Colony-Stimulating Factor
rhEGF	Recombinant Human Epidermal Growth Factor
HGF	Hepatocyte Growth Factor
ANG-1	Angiopoietin-1
NMA	Network Meta-Analysis
PU	Pressure Ulcer
PUSH	Pressure Ulcer Scale for Healing
GM-CSF	Granulocyte–Macrophage Colony-Stimulating Factor
VLU	Venous Leg Ulcer
PRF	Platelet-Rich Fibrin
